# Metabolic Characteristics and Flavonoid Differences in Peel and Flesh of Three Kumquat Cultivars Based on Non-Targeted Metabolomics

**DOI:** 10.3390/foods15213934

**Published:** 2026-11-03

**Authors:** Chanchan Huang, Lilan Lyu, Chengwu Zou, Yongfeng Zhao, Jinjie Yao, Yuye Chen, Jin Xie, Lulu Si, Ganlin Chen

**Affiliations:** 1 Guangxi Subtropical Crops Research Institute, Guangxi Academy of Agricultural Sciences, Nanning 530001, China; 2 Quality and Testing Center of Subtropical Fruit and Vegetable of Ministry of Agriculture and Rural Affairs, Nanning 530001, China; 3 Key Laboratory of Quality and Safety Control for Subtropical Fruit and Vegetable, Ministry of Agriculture and Rural Affairs, Nanning 530001, China; 4 Guangxi Key Laboratory of Quality and Safety Control for Subtropical Fruits, Nanning 530001, China; 5 Laboratory of Quality Risk Assessment for Agro-Products of Ministry of Agriculture and Rural Affairs, Nanning 530001, China; 6 College of Agriculture, Guangxi University, Nanning 530001, China; 7 Technical Center of Nanning Customs District, Nanning 530022, China

**Keywords:** untargeted metabolomics, kumquat, flavonoids

## Abstract

Untargeted metabolomics based on ultra-high performance liquid chromatography-tandem mass spectrometry (UPLC-MS/MS) was used to explore metabolite differences in the peel and flesh tissues of three Rong’an kumquat cultivars. A total of 5043 metabolites were detected. Principal component analysis (PCA) revealed clear separation of metabolic profiles among different cultivars. Significantly altered metabolites were screened with thresholds of VIP > 1 and |Log_2_FC| ≥ 1.0. A total of 193 flavonoid metabolites were annotated, among which 84 flavones and 28 flavonols were the dominant subclasses. Flavonoid profiles exhibited significant cultivar- and tissue-specific differences. Huapi kumquat (HP) and Cuimi kumquat (CM) showed tissue-specific anthocyanin accumulation, with anthocyanins enriched mainly in peel tissues, while Youpi kumquat (YP) accumulated abundant chalcones and flavanones. The flavonoid metabolic variation in peel tissues was greater than that in flesh tissues. 2′,6′-dihydroxy-4,4′-dimethoxychalcone (with log_2_FC up to 8.44) and xanthohumol were identified as candidate metabolic markers. KEGG pathway enrichment analysis further revealed distinct tissue-biased metabolic features. Specifically, cultivar-related metabolic variation in peel was associated with diterpenoid and lipid metabolic pathways, whereas flesh metabolic divergence was linked to indole alkaloid biosynthesis, histidine metabolism, and folate biosynthesis. This study systematically elucidated the metabolic characteristics and flavonoid differences in the peel and flesh of three kumquat cultivars, providing candidate metabolic markers and theoretical support for the evaluation and utilization of kumquat germplasm resources.

## 1. Introduction

Kumquat (*Fortunella crassifolia* Swingle), a member of the genus *Fortunella* in the family *Rutaceae*, is a golden-yellow citrus fruit with an oval or round shape. It is widely used for both medicinal and edible purposes, and its peel and flesh are both edible [1]. Kumquat is extensively cultivated and distributed worldwide, particularly in southeastern China [2]. The fruit is sweet and fragrant and can be consumed directly with its peel. It is rich in nutrients such as vitamins [3], minerals [4], and organic acids [4], as well as secondary metabolites including flavonoids [5] and phenolic acids [6]. Kumquat exhibits effects in invigorating the stomach, regulating qi, relieving cough, and eliminating phlegm. Moreover, kumquat fruit extracts possess various physiological functions, such as antioxidant and anti-inflammatory activities [3,7].

In recent years, metabolomic studies on citrus fruits have become a research hotspot. Kim et al. [8] analyzed the total phenolic content (TPC), total carotenoid content (TCC), antioxidant activity, and metabolite profiles in Citrus unshiu fruit flesh at different stages of fruit development and evaluated their correlations. Wang et al. [9] used UHPLC-QQQ-MS-based metabolomics to analyze metabolite profiles and relative contents in the peels of huanglongbing (HLB)-infected *Citrus reticulata* cv. ‘Shatangju’ fruits with different peel colors. Li et al. [10] distinguished *Citrus reticulata* Blanco and *Citrus reticulata* ‘Chachi’ using a GC-MS-based metabolomics approach. Peng et al. [11] analyzed the primary metabolites and phenolic acids in five citrus cultivars using liquid chromatography-tandem mass spectrometry (LC-MS/MS). Nevertheless, metabolomic investigations on kumquat remain relatively scarce compared with other citrus varieties. Li et al. [12] performed non-targeted metabolomic analysis of three kumquat genotypes, namely Changshou (FOT), Liuyang (FCSLY), and Huapi (FCSHP), and identified a total of 1719 metabolites. Flavonoid metabolites were more abundant in kumquat peels than in seeds. Ma et al. [13] integrated metabolomic profiling and gene expression analysis to characterize 201 differential compounds, including 65 flavonoids and 37 lipids. Most of the differential flavonoids were glycosylated with hexoside and accumulated at higher levels in the peel but at lower levels in the flesh of ‘Huapi’ kumquat compared with the wild-type fruit. These previous studies have laid a solid foundation for kumquat metabolite research. However, existing metabolomic studies have mainly focused on ‘Huapi’ and ‘Youpi’ kumquats, while the metabolic profiles of the peel and flesh of ‘Cuimi’ kumquat remain largely unexplored. Accordingly, the present study performed untargeted metabolomic analysis of three commercial kumquat cultivars ‘Huapi’, ‘Youpi’, and ‘Cuimi’), primarily aiming to elucidate cultivar-specific metabolic differences in the peel and flesh tissues.

Kumquat cultivation in Rong’an, Guangxi Zhuang Autonomous Region, China, spans over 300 years [2]. Rong’an County serves as China’s core kumquat production base and possesses multiple superior cultivars, namely ‘Youpi Kumquat’, ‘Huapi Kumquat’, and ‘Cuimi Kumquat’. ‘Youpi Kumquat’, the earliest cultivated local cultivar, is recognized as the first-generation kumquat. Derived from a seedling mutation of ‘Youpi Kumquat’, ‘Huapi Kumquat’ is categorized as the second-generation cultivar, which features higher sweetness and milder pungency compared with ‘Youpi Kumquat’. ‘Cuimi Kumquat’, the third-generation Rong’an kumquat, was developed through artificial breeding and selection from a natural bud sport of ‘Huapi Kumquat’ [14]. In this study, these three typical cultivars were selected as experimental materials. Untargeted metabolomics based on UPLC-MS/MS was applied to characterize tissue-specific metabolic profiles and flavonoid differences between the peel and flesh of kumquat, aiming to provide theoretical support for the deep processing and targeted breeding of kumquat.

## 2. Materials and Methods

### 2.1. Plant Materials

The experimental materials included peel and flesh samples from three kumquat cultivars: (1) ‘Youpi Kumquat’ (abbreviation: YP; peel: YPP, flesh: YPF), (2) ‘Huapi Kumquat’ (abbreviation: HP; peel: HPP, flesh: HPF), and (3) ‘Cuimi Kumquat’ (abbreviation: CM; peel: CMP, flesh: CMF). Mature fruits of three kumquat cultivars were harvested from a commercial orchard in Rong’an County, Liuzhou, Guangxi Zhuang Autonomous Region, China (109°35′32″ E, 25°18′14″ N). For each cultivar, fruits were randomly collected from the outer canopy of at least three independent trees with uniform growth vigor. More than 40 intact and healthy fruits were harvested from each tree. All harvested fruits of the same cultivar were pooled and immediately transported to the laboratory in insulated ice boxes. The peel and flesh tissues of fruits from each cultivar were manually separated. The peel of each cultivar was divided into three equal subsamples to form three biological replicates, and the flesh was aliquoted into three biological replicates in the same manner. The isolated tissues were immediately snap-frozen in liquid nitrogen and stored at −80 °C in an ultra-low temperature freezer for subsequent untargeted metabolomic analysis.

### 2.2. Metabolite Extraction

Kumquat peel and flesh samples were freeze-dried using a vacuum freeze dryer (SCIENTZ-100F, Ningbo Scientz Biotechnology Co., Ltd., Ningbo, China). The dried samples were ground into powder using a tissue grinder (MM 400, Retsch GmbH, Haan, Germany) at 30 Hz for 1.5 min. Briefly, 50 mg of sample powder was weighed using an electronic balance (MS105DU, Mettler Toledo, Zurich, Switzerland), and 1200 μL of pre-cooled (−20 °C) 70% aqueous methanol (*v*/*v*, 70% methanol in ultrapure water) was added. The mixture was vortexed for 30 s every 30 min, for six cycles in total. After centrifugation at 12,000 rpm for 3 min, the supernatant was collected. The supernatant was filtered through a 0.22 μm microporous membrane and transferred into an injection vial for subsequent UPLC-MS/MS analysis.

### 2.3. UPLC Conditions

All samples were analyzed using a Shimadzu LC-30A UPLC system (Shimadzu Corporation, Kyoto, Japan) coupled with an AB SCIEX TripleTOF 6600 mass spectrometer (SCIEX, Foster City, CA, USA) according to the predefined instrument acquisition program. The analytical conditions were as follows. The UPLC separation was performed on a Waters ACQUITY UPLC HSS T3 column (Waters Corporation, Milford, MA, USA; 1.8 µm, 2.1 mm × 100 mm) maintained at 40 °C. The flow rate was set at 0.40 mL/min, and the injection volume was 4 μL. The mobile phase consisted of water containing 0.1% formic acid (Phase A) and acetonitrile containing 0.1% formic acid (Phase B). The gradient elution program was set as follows: initial condition at 0.0 min, 95% A and 5% B; linearly changed to 35% A and 65% B at 5.0 min; linearly changed to 1% A and 99% B at 6.0 min, and held until 7.5 min; then returned to 95% A and 5% B within 0.1 min (7.6 min), and maintained until 10.0 min for column re-equilibration.

### 2.4. MS Conditions

Mass spectrometric measurements were performed on an AB SCIEX TripleTOF 6600 mass spectrometer equipped with a time-of-flight (TOF) mass analyzer. Data acquisition was carried out in information-dependent acquisition (IDA) mode using Analyst TF 1.7.1 software (Sciex, Concord, ON, Canada). Separate analytical runs were performed for electrospray ionization positive-ion (ESI+) and negative-ion (ESI−) modes. The ion-source parameters were set as follows: ion spray voltage, 5000 V for ESI+ and −4000 V for ESI−; ion source temperature, 550 °C; ion source gas 1 (GAS1), 50 psi; ion source gas 2 (GAS2), 60 psi; curtain gas (CUR), 35 psi; declustering potential (DP), 60 V for ESI+ and −60 V for ESI−. For the TOF-MS1 full-scan experiment, the collision energy was 10 V (ESI+)/−10 V (ESI−), with a mass range of 50–1250 Da. For product-ion (TOF-MS2) scans, the collision energy was 30 V (ESI+)/−30 V (ESI−), with a collision energy spread of 15 V, and the mass range was set to 25–1250 Da. The acquisition duration for each injection was 10 min. Mass-axis calibration was conducted with the manufacturer-supplied calibration solution prior to each analytical batch to ensure mass accuracy. Datasets obtained from positive-ion-mode and negative-ion-mode acquisitions were processed independently and were not combined for metabolite identification and quantification.

### 2.5. Data Processing and Analysis

The original data file, acquired by LC-MS, was converted into mzXML format through ProteoWizard software (v3.0). Peak extraction, peak alignment, and retention-time correction were performed using the XCMS program. The “SVR” method was used for peak-area correction. Peaks with a detection rate lower than 50% in each sample group were discarded. Subsequently, metabolite annotation was performed by searching against the laboratory’s in-house spectral library, integrated public databases, AI-assisted spectral library, and the metDNA tool.

The precursor-ion mass-matching tolerance was set to ±50 mDa for both positive- and negative-ion modes. Experimentally acquired MS/MS fragment spectra were matched against reference spectra, and a spectral similarity score threshold of ≥0.7 was adopted for putative annotation. Annotation confidence was classified according to the Metabolomics Standards Initiative (MSI) criteria [15].

Unsupervised PCA (principal component analysis) was performed using the stats function prcomp in R (version 4.1.2, www.r-project.org). The data were unit variance scaled before unsupervised PCA to eliminate bias caused by large differences in signal magnitude among metabolites. The HCA (hierarchical cluster analysis) results of samples and metabolites were presented as heatmaps with dendrograms, while Pearson correlation coefficients (PCC) between samples were calculated by the cor function in R and presented only as heatmaps. Both HCA and PCC were carried out using the R package ComplexHeatmap (version 2.9.4). For HCA, normalized signal intensities of metabolites (unit variance scaling) were visualized as a color spectrum. For two-group analysis, significantly altered metabolites were determined by variable importance in projection (VIP > 1) and absolute Log_2_fold change (|Log_2_FC| ≥ 1.0). VIP values were obtained from the orthogonal partial least squares discriminant analysis (OPLS-DA) model. OPLS-DA score plots and permutation plots were generated using the R package MetaboAnalystR (version 1.0.1). The data were log-transformed (log_2_) and mean-centered before OPLS-DA. To avoid overfitting, a permutation test (200 permutations) was performed. Identified metabolites were annotated using the KEGG Compound database (http://www.kegg.jp/kegg/compound/ (accessed on 29 February 2024)), and annotated metabolites were then mapped to the KEGG Pathway database (http://www.kegg.jp/kegg/pathway.html (accessed on 29 February 2024)). Significantly enriched pathways were identified based on the *p*-value of a hypergeometric test for a given list of metabolites.

## 3. Results

### 3.1. Multivariate Statistical Analysis

To clarify the metabolic differences between the peel and flesh tissues across three Rong’an kumquat cultivars, UPLC-MS/MS-based untargeted metabolomics was used to determine the metabolites in the peel and flesh of kumquat. A total of 5043 LC-MS features were detected in the three kumquat samples (Table S1), including 1498 amino acids and derivatives, 607 organic acids, 589 benzene and substituted derivatives, 227 alkaloids, 193 flavonoids, 186 alcohols and amines, 184 heterocyclic compounds, 145 glycerophospholipids (GP), 143 phenolic acids, 139 nucleotides and derivatives, 107 terpenoids, 89 lignans and coumarins, 79 lipids, 65 fatty acyls (FA), 40 glycerolipids (GL), 40 steroids, 17 quinones, 9 sphingolipids (SL), 7 tryptamines, cholines and pigments, 5 tannins, and 674 others.

Quality control (QC) samples were prepared by pooling equal aliquots of extracts from all individual samples to monitor the stability and reproducibility of sample pretreatment and instrument detection. The LC-MS quality control results under both positive and negative ion modes are shown in Figure S1, demonstrating stable instrument signals, negligible sample carryover, high correlation among QC samples, and low variation in metabolite signals throughout the analytical run. Based on MSI classification criteria, all detected features were annotated and graded. Among them, 1170 features were assigned to MSI Level 2, and 3873 features were classified as MSI Level 3. Detailed annotation information for all detected features, including mass error, MS/MS similarity score, identification level, molecular formula, adduct type, and corresponding database IDs, is summarized in Table S1.

**Figure 1:**
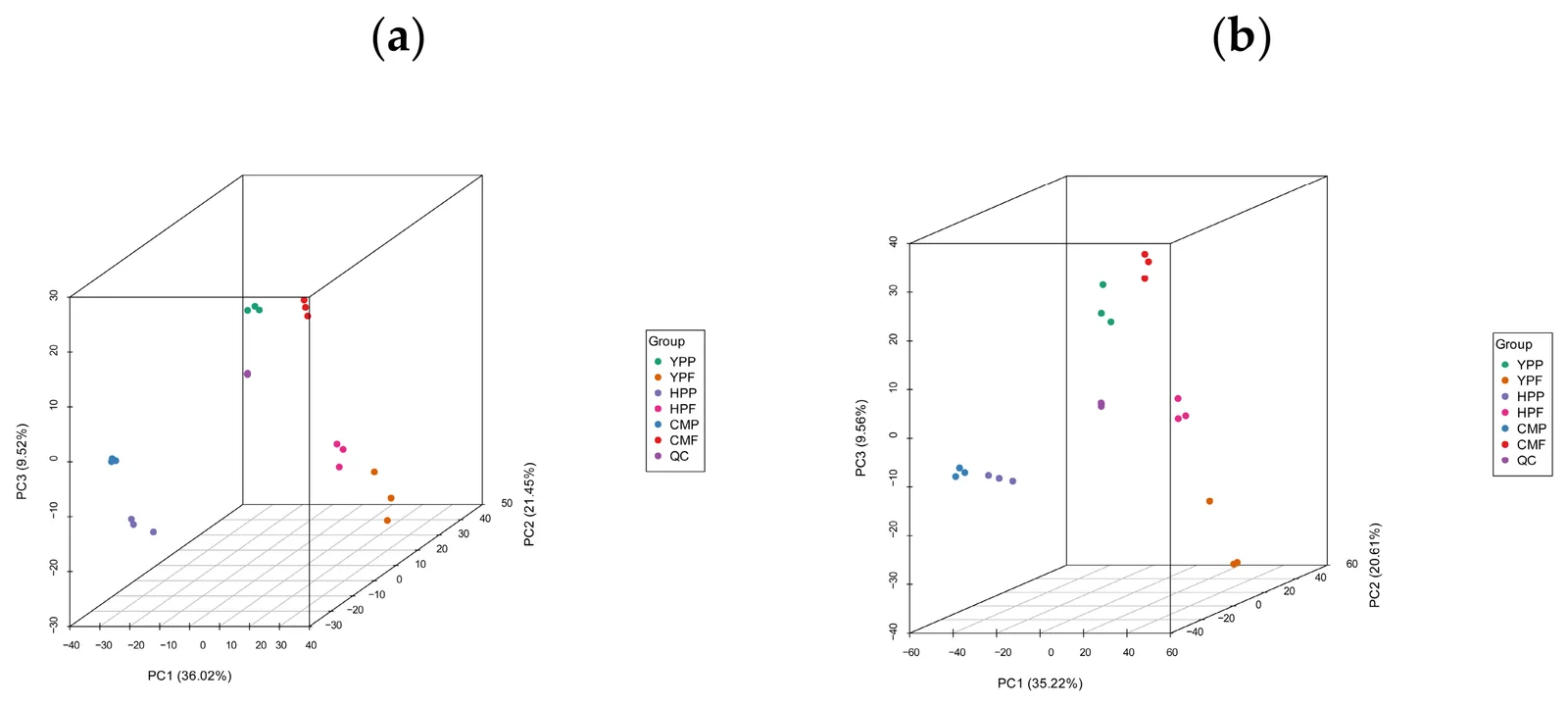
Three-dimensional PCA score plots of kumquat peel and flesh samples. (**a**) Positive ionization mode; (**b**) Negative ionization mode. PC1, PC2, and PC3 represent the first, second, and third principal components, respectively. The percentage values indicate the proportion of total variance explained by each principal component. QC, quality control samples.

To further explore the global metabolic differences among the three kumquat cultivars, principal component analysis (PCA) was performed on samples of the three kumquat cultivars together with quality control (QC) samples (Figure 1). Three-dimensional PCA score plots were constructed to visualize sample distributions across PC1, PC2, and PC3. Under positive ionization mode, PC1, PC2, and PC3 explained 36.02%, 21.45%, and 9.52% of the total variance, respectively. Under negative ionization mode, PC1, PC2, and PC3 explained 35.22%, 20.61%, and 9.56% of the total variance, respectively. PCA results indicated significant differences among the three kumquat cultivars. The QC samples were located at the center of the PCA score plot, and the three biological replicates within each group clustered closely together, while samples from different cultivars or tissues were clearly separated from each other. This demonstrated that the experimental method had good reproducibility and reliability. In addition, the peel and flesh samples of YP showed significant differences from those of the other two kumquat cultivars, whereas HP and CM exhibited certain similarities. PC3 accounted for a moderate proportion of the total variation and mainly reflected minor intra-group variation rather than the major inter-cultivar metabolic differences captured by PC1 and PC2.

### 3.2. Identification of Significantly Altered Metabolites

**Figure 2:**
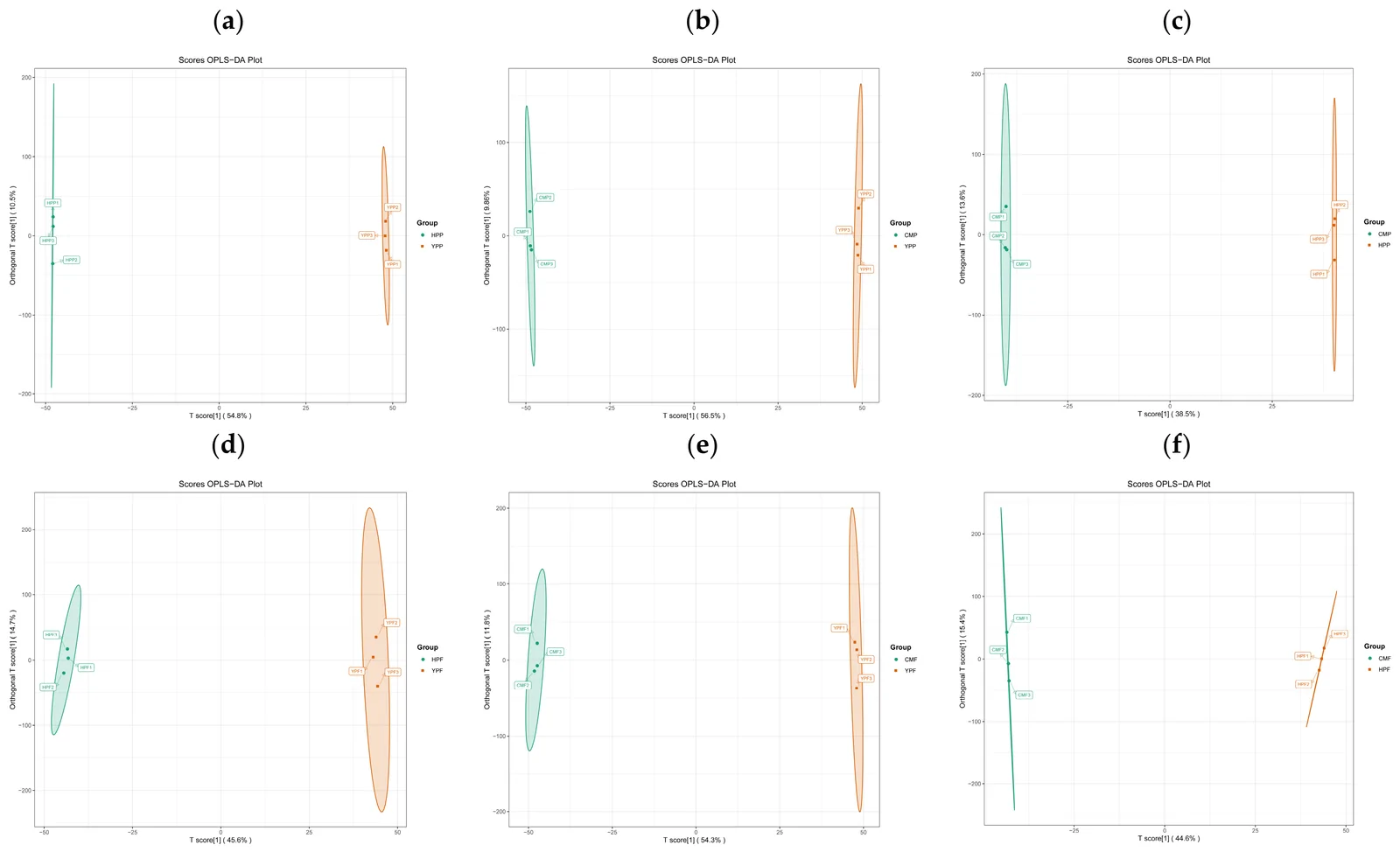
Analysis of significantly altered metabolites based on OPLS-DA score plots. (**a**) HPP vs. YPP; (**b**) CMP vs. YPP; (**c**) CMP vs. HPP; (**d**) HPF vs. YPF; (**e**) CMF vs. YPF; (**f**) CMF vs. HPF.

OPLS-DA is a multivariate statistical method for supervised pattern recognition, which can effectively eliminate irrelevant influences and thereby screen significantly altered metabolites [16]. OPLS-DA was used to analyze the peel and flesh of YP, HP and CM kumquat cultivars, and the corresponding score plots were constructed (Figure 2). In this model, R^2^X and R^2^Y represent the explanatory power of the established model for the X and Y matrices, respectively, and Q^2^ indicates the predictive ability of the model. HPP vs. YPP (Q^2^ = 0.961, R^2^X = 0.653, R^2^Y = 1; Figure 2a), CMP vs. YPP (Q^2^ = 0.969, R^2^X = 0.663, R^2^Y = 1; Figure 2b), CMP vs. HPP (Q^2^ = 0.877, R^2^X = 0.521, R^2^Y = 1; Figure 2c), HPF vs. YPF (Q^2^ = 0.901, R^2^X = 0.604, R^2^Y = 1; Figure 2d), CMF vs. YPF (Q^2^ = 0.943, R^2^X = 0.661, R^2^Y = 1; Figure 2e), CMF vs. HPF (Q^2^ = 0.892, R^2^X = 0.600, R^2^Y = 1; Figure 2f). In all comparison groups, R^2^Y = 1 and Q^2^ values were all greater than 0.85, suggesting that the established OPLS-DA models were stable and reliable for further screening of significantly altered metabolites. A permutation test (200 permutations) was performed to validate each OPLS-DA model and exclude overfitting. The permutation test plots are provided in Figure S2. All permutation tests yielded *p* < 0.005, confirming that the OPLS-DA models were statistically significant and free from overfitting.

**Figure 3:**
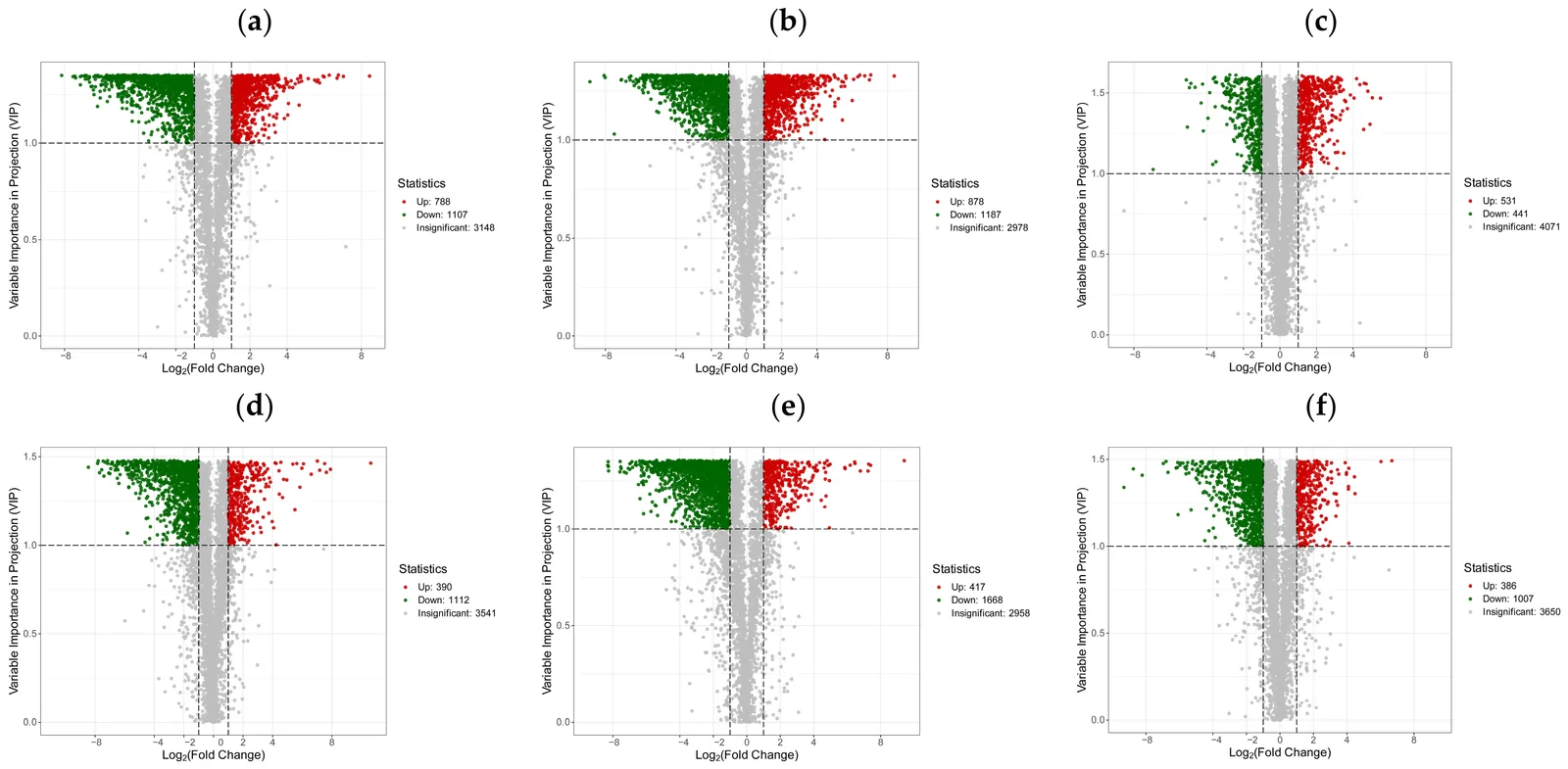
Volcano plots of significantly altered metabolites in peel and flesh samples. Red dots indicate up-regulated metabolites, and green dots indicate down-regulated metabolites. The horizontal dashed line represents VIP = 1.0, and the two vertical dashed lines represent |Log_2_FC| = 1. (**a**) HPP vs. YPP; (**b**) CMP vs. YPP; (**c**) CMP vs. HPP; (**d**) HPF vs. YPF; (**e**) CMF vs. YPF; (**f**) CMF vs. HPF.

To comprehensively investigate the differences among metabolites, we screened significantly altered metabolites from all identified compounds. Significantly altered metabolites were screened based on fold change (FC) and variable importance in projection (VIP) values, using the criteria of VIP > 1 and |Log_2_FC| ≥ 1.0. The screening results were visualized in volcano plots (Figure 3). The results showed 1895 significantly altered metabolites between HPP and YPP, among which 788 were up-regulated and 1107 were down-regulated. Between CMP and YPP, 2065 significantly altered metabolites were identified, of which 878 were up-regulated and 1187 were down-regulated. Between CMP and HPP, 972 significantly altered metabolites were detected, including 531 up-regulated and 441 down-regulated metabolites. Between HPF and YPF, 1502 significantly altered metabolites were observed, with 390 up-regulated and 1112 down-regulated metabolites. Between CMF and YPF, 2085 significantly altered metabolites were found, among which 417 were up-regulated and 1668 were down-regulated. Between CMF and HPF, 1393 significantly altered metabolites were identified, including 386 up-regulated and 1007 down-regulated metabolites.

### 3.3. Differential Accumulation of Flavonoids

The plant kingdom is the main dietary source of flavonoids for humans, and, in particular, citrus fruits possess a rich and varied flavonoid pool [17]. Previous studies have found that kumquat fruits exhibit potent antioxidant activity, antimicrobial activity, liver-protective activity, and anticancer activity [18]. Therefore, investigating the flavonoid metabolites in kumquats is of great significance. A total of 193 flavonoid metabolites were identified in this study, including 84 flavones, 28 flavonols, 23 isoflavones, 13 anthocyanins (only one anthocyanidin aglycone was detected among them), 12 chalcones, 9 flavanones, 4 flavanols, 1 flavanonol, and 19 other flavonoids (Table S2). As shown in Table S2, flavones represented the most abundant subclass, followed by flavonols. Among these metabolites, kaempferol 3-O-β-D-glucopyranosyl-7-O-α-L-rhamnopyranoside and vitexin 2″-O-rhamnoside exhibited the highest relative abundance in kumquat fruits.

**Figure 4:**
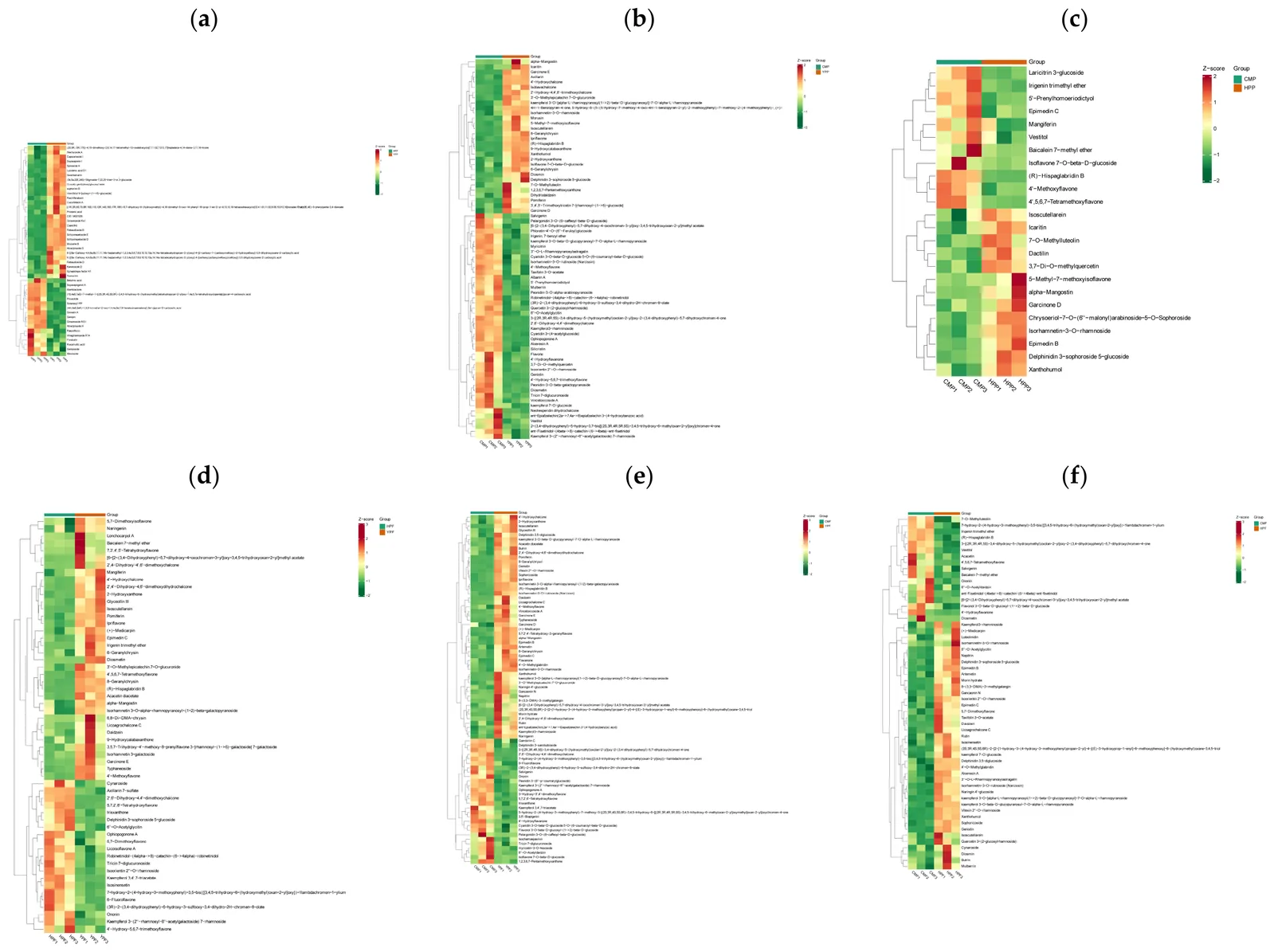
Cluster heatmaps of significantly altered flavonoid metabolites. (**a**) HPP vs. YPP; (**b**) CMP vs. YPP; (**c**) CMP vs. HPP; (**d**) HPF vs. YPF; (**e**) CMF vs. YPF; (**f**) CMF vs. HPF. Each column corresponds to a single sample, and each row corresponds to one metabolite. The color gradient from red to green shows metabolite abundance decreasing from high to low.

To further clarify the specific differences in flavonoid metabolism among varieties and between tissues, pairwise metabolite analyses were performed on the peels and flesh of three kumquat cultivars (Figure 4). In the HPP vs. YPP comparison, a total of 73 flavonoid metabolites were identified as differentially accumulated (VIP > 1), of which 46 were up-accumulated and 27 were down-accumulated in HPP relative to YPP (Figure 4a; Table S3). Flavones represented the most abundant subclass (28), with 18 up-accumulated and 10 down-accumulated metabolites. All six anthocyanins were up-accumulated in HPP, with no down-accumulated members. Peonidin-3-O-β-galactopyranoside and cyanidin 3-O-β-D-(caffeoyl)-sambubioside showed particularly strong up-accumulation. Among the six differentially accumulated chalcones, four metabolites (xanthohumol, 2′-hydroxy-4,4′,6′-trimethoxychalcone, 4′-hydroxychalcone, and isobavachalcone) were down-accumulated in HPP. Meanwhile, 2′,6′-dihydroxy-4,4′-dimethoxychalcone exhibited an exceptionally high fold change (log_2_FC = 8.44) and was strongly enriched in HPP.

In the CMP vs. YPP comparison, a total of 74 flavonoid metabolites were identified as differentially accumulated. Flavones represented the most abundant subclass (29), with 20 up-accumulated and 9 down-accumulated metabolites. Among the six anthocyanins, five were up-accumulated in CMP, while delphinidin 3-sophoroside 5-glucoside was down-accumulated. Seven chalcones exhibited differential accumulation. Four chalcones, including xanthohumol, 2′-hydroxy-4,4′,6′-trimethoxychalcone, 4′-hydroxychalcone, and isobavachalcone showed higher abundance in YPP. By contrast, three chalcones were enriched in CMP, among which 2′,6′-dihydroxy-4,4′-dimethoxychalcone displayed an extremely large fold change (log_2_FC = 8.38) (Figure 4b; Table S3).

In the CMP vs. HPP comparison, a total of 24 flavonoid metabolites were identified as differentially accumulated, among which flavones represented the most abundant subclass (10 members). Only one anthocyanin (delphinidin 3-sophoroside 5-glucoside) and one chalcone (xanthohumol) showed differential accumulation, and both were down-accumulated in CMP relative to HPP. Among the five differentially accumulated flavonols, four, including isorhamnetin-3-O-rhamnoside, 3,7-di-O-methylquercetin, icaritin, and epimedin B, were down-accumulated in CMP relative to HPP (Figure 4c; Table S3).

In the HPF vs. YPF comparison, a total of 57 flavonoid metabolites were identified as differentially accumulated, of which 21 were up-accumulated and 36 were down-accumulated in HPF relative to YPF. Flavones represented the most abundant subclass (27), with 11 up-accumulated and 16 down-accumulated metabolites (Figure 4d; Table S3). Within the anthocyanin subclass, only delphinidin 3-sophoroside 5-glucoside was up-accumulated. Among the five chalcones with differential accumulation, only 2′,6′-dihydroxy-4,4′-dimethoxychalcone was up-accumulated (log_2_FC = 7.91). By contrast, isoflavones, flavanones, and most chalcones exhibited higher abundance in YPF. Isoflavones including irigenin trimethyl ether and (R)-hispaglabridin B, as well as the flavone isoscutellarein, exhibited high relative abundance in YPF.

In the CMF vs. YPF comparison, a total of 78 flavonoid metabolites were identified as differentially accumulated, of which 28 were up-accumulated and 50 were down-accumulated in CMF relative to YPF. Flavones represented the most abundant subclass (32), with 13 up-accumulated and 19 down-accumulated metabolites (Figure 4e; Table S3). Among the five differentially accumulated anthocyanins, four, including pelargonidin 3-O-(6-caffeoyl-β-D-glucoside) and peonidin 3-(6″-p-coumarylglucoside), were up-accumulated in CMF, whereas delphinidin 3,5-diglucoside was down-accumulated. Among the six chalcones with differential accumulation, five were down-accumulated in CMF, whereas 2′,6′-dihydroxy-4,4′-dimethoxychalcone exhibited a large fold change (log_2_FC = 7.25) and was strongly up-accumulated in CMF. Both flavanones (naringenin and flavanone) were down-accumulated in CMF.

In the CMF vs. HPF comparison, a total of 57 flavonoid metabolites were identified as differentially accumulated, of which 17 were up-accumulated and 40 were down-accumulated in CMF relative to HPF. Flavones represented the most abundant subclass (27), with 10 up-accumulated and 17 down-accumulated metabolites (Figure 4f; Table S3). All detected anthocyanins (delphinidin 3-sophoroside 5-glucoside and delphinidin 3,5-diglucoside), the aglycone luteolinidin, and both chalcones (xanthohumol and licoagrochalcone C) were down-accumulated in CMF relative to HPF. Xanthohumol and the isoflavone genistin were the most markedly down-accumulated metabolites, whereas irigenin trimethyl ether, baicalein 7-methyl ether, and diosmetin were strongly up-accumulated in CMF.

Collectively, the pairwise comparisons revealed obvious tissue- and cultivar-dependent variation in kumquat flavonoid profiles. Generally, peel tissues possessed more flavonoid metabolites with differential accumulation than flesh tissues. HP- and CM-derived samples (both peel and flesh) exhibited relatively similar flavonoid patterns, while substantial metabolic divergence was observed between the HP and CM samples and the YP samples. The chalcone 2′,6′-dihydroxy-4,4′-dimethoxychalcone stood out as a highly accumulated metabolite in HP and CM tissues across both peel and flesh, whereas several other chalcone members preferentially accumulated in YP tissues.

### 3.4. KEGG Enrichment Analysis of Three Kumquat Cultivars

**Figure 5:**
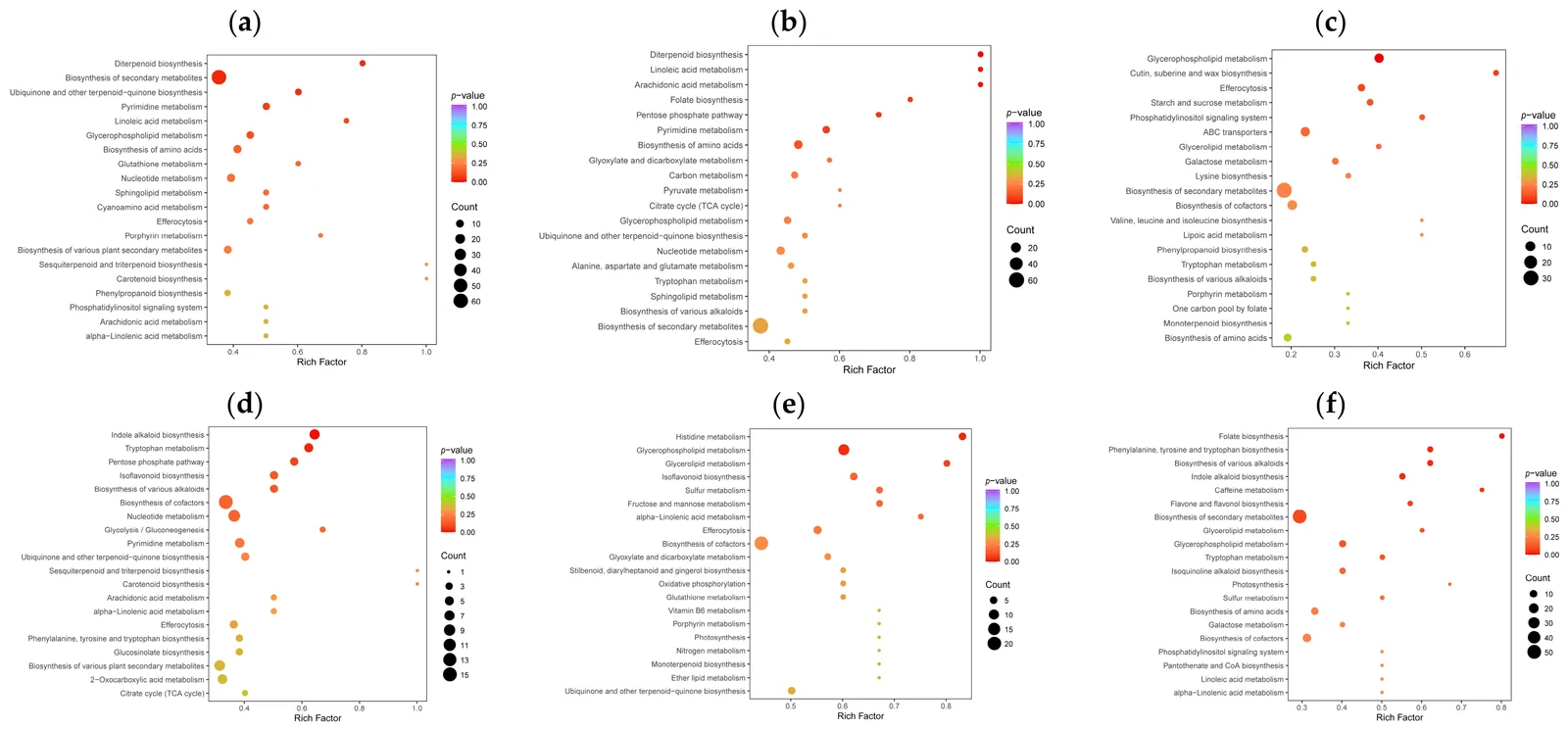
KEGG pathway enrichment analysis of significantly altered metabolites from pairwise comparisons between the peel and flesh of each cultivar. (**a**) HPP vs. YPP; (**b**) CMP vs. YPP; (**c**) CMP vs. HPP; (**d**) HPF vs. YPF; (**e**) CMF vs. YPF; (**f**) CMF vs. HPF. Each bubble in the plot represents a metabolic pathway, and its horizontal position (abscissa) and bubble size jointly indicate the pathway impact factor. A larger bubble size indicates a larger impact factor. The bubble colors represent the *p*-values of the enrichment analysis, with darker colors indicating a higher degree of enrichment.

The Kyoto Encyclopedia of Genes and Genomes (KEGG) database is the main public database of metabolic pathways, which can be used in studies of gene expression information and metabolite accumulation in a general network [19]. In this study, KEGG pathway enrichment analysis was performed using differentially accumulated metabolites from peel and flesh samples of each cultivar, and major enriched pathways were visualized with bubble charts (Figure 5). KEGG enrichment results for peel tissues of three kumquat cultivars are summarized in Table S4 and Figure 5a–c.

For HPP vs. YPP (Figure 5a), the top five enriched pathways based on altered metabolite accumulation were diterpenoid biosynthesis (ko00904), biosynthesis of secondary metabolites (ko01110), ubiquinone and other terpenoid-quinone biosynthesis (ko00130), pyrimidine metabolism (ko00240), and linoleic acid metabolism (ko00591). These pathways are mainly involved in secondary metabolism, lipid metabolism, energy metabolism, and nucleic acid metabolism. Within diterpenoid biosynthesis (ko00904), which showed the strongest enrichment signal, one metabolite (stevioside) exhibited higher relative abundance in HPP, whereas three metabolites (geranylgeraniol, geranyllinalool, and geranylgeranyl diphosphate) showed lower relative abundance. These observations indicated altered relative accumulation of diterpenoid-related metabolites.

For CMP vs. YPP (Figure 5b), the top five enriched pathways were diterpenoid biosynthesis (ko00904), linoleic acid metabolism (ko00591), arachidonic acid metabolism (ko00590), folate biosynthesis (ko00790), and pentose phosphate pathway (ko00030). Diterpenoid biosynthesis (ko00904) showed the most prominent enrichment and contained five differentially accumulated metabolites. Among them, four metabolites (abietic acid, geranylgeraniol, geranyllinalool, and geranylgeranyl diphosphate) displayed lower relative abundance in CMP, while only stevioside showed higher relative abundance. These results reflected shifts in the relative abundance of metabolites related to diterpenoids and secondary-metabolite modification between CMP and YPP.

For CMP vs. HPP (Figure 5c), efferocytosis (ko04148) was excluded from the main-text description because it represents a mammalian-specific pathway with limited biological relevance to plants. Accordingly, the top five plant-relevant enriched pathways were glycerophospholipid metabolism (ko00564), cutin, suberine, and wax biosynthesis (ko00073), starch and sucrose metabolism (ko00500), phosphatidylinositol signaling system (ko04070), and ABC transporters (ko02010). Glycerophospholipid metabolism (ko00564) exhibited the strongest enrichment signal. Within this pathway, six phospholipid-related metabolites showed lower relative abundance in CMP, whereas two metabolites (1-palmitoyl-2-oleoyl-sn-glycero-3-phosphate and LPC(20:1)) showed higher relative abundance. These patterns indicated differences in the relative abundance of lipid-related metabolite pools between CMP and HPP.

KEGG enrichment results for flesh tissues of three kumquat cultivars are summarized in Table S4 and Figure 5d–f. For HPF vs. YPF (Figure 5d), the top five enriched pathways were indole alkaloid biosynthesis (ko00901), tryptophan metabolism (ko00380), pentose phosphate pathway (ko00030), isoflavonoid biosynthesis (ko00943), and biosynthesis of various alkaloids (ko00996). Indole alkaloid biosynthesis (ko00901) showed the strongest enrichment signal; harmaline was more abundant in HPF, while the other six pathway-associated metabolites displayed lower relative abundance. These results pointed to pronounced differences in relative metabolite abundance within the indole alkaloid biosynthesis pathway between HPF and YPF.

For CMF vs. YPF (Figure 5e), the top five enriched pathways were histidine metabolism (ko00340), glycerophospholipid metabolism (ko00564), glycerolipid metabolism (ko00561), isoflavonoid biosynthesis (ko00943), and sulfur metabolism (ko00920). Histidine metabolism (ko00340) was the most prominently enriched pathway and contained five differentially accumulated metabolites. Only L-glutamic acid showed higher relative abundance in CMF, whereas four metabolites (imidazoleacetic acid, ergothioneine, urocanic acid, and imidazolepropionic acid) exhibited lower relative abundance. The data indicated substantial differences in metabolite accumulation associated with histidine metabolism between CMF and YPF.

For CMF vs. HPF (Figure 5f), the top five enriched pathways were folate biosynthesis (ko00790), phenylalanine, tyrosine and tryptophan biosynthesis (ko00400), biosynthesis of various alkaloids (ko00996), indole alkaloid biosynthesis (ko00901), and caffeine metabolism (ko00232). Folate biosynthesis (ko00790) showed the strongest enrichment signal and included four differentially accumulated metabolites: chorismic acid, dihydrofolic acid, sepiapterin, and 7,8-dihydromethanopterin. All four metabolites exhibited lower relative abundance in CMF, with no up-accumulated compounds detected. Notably, folic acid serves as an essential coenzyme precursor participating in purine, pyrimidine, and amino acid biosynthesis [20].

## 4. Discussion

Previous studies have shown that flavonoids are involved in multiple biological functions and have important health-related effects, such as antioxidant activity [21], anti-diabetic effects [22], anti-inflammatory effects [23], and anti-hypertensive effects [24]. In this study, a total of 193 flavonoid metabolites were identified in kumquat fruits, predominantly consisting of flavones and flavonols. As shown in Table S2, flavones represented the most abundant subclass, followed by flavonols. Among these metabolites, kaempferol 3-O-β-D-glucopyranosyl-7-O-α-L-rhamnopyranoside and vitexin 2″-O-rhamnoside exhibited the highest relative abundance in kumquat fruits. Kaempferol is a typical flavonoid with anticancer, anti-inflammatory, and antioxidant activities [25]. Similarly to the results of this study, Kawaii et al. [26] quantified flavonoids in various citrus cultivars, including kumquat (*Fortunella japonica*, *Fortunella margarita,* and *Fortunella crassifolia*), with samples extracted using a mixture of methanol and dimethyl sulfoxide. The major quantified flavonoids in the edible part of kumquat included kaempferol and other flavonoid compounds. Cho et al. [27] investigated the chemical constituents of the fruit peels of *Fortunella japonica* Swingle. Using a variety of chromatographic methods for isolation and purification, they identified kaempferide 3-O-rhamnopyranoside from the peel. Vitexin is an apigenin flavone glycoside. It exhibits multiple pharmacological activities, including antioxidant, anti-inflammatory, anticancer, antinociceptive, and neuroprotective effects [28].

Consistent with pairwise comparison results, peel tissues contained more differentially accumulated flavonoids than flesh tissues, indicating that peel serves as the primary site for flavonoid accumulation in kumquat fruit [3,13,29]. This trend agreed well with the PCA findings. A reasonable explanation is that CM was artificially selected from HP, which may partly account for their similar flavonoid profiles. By contrast, distinct metabolic divergence was observed between the HP–CM group and YP samples. Within peel tissues, flavones and anthocyanins largely contributed to the metabolic differences between HPP and YPP. Notably, all six anthocyanins were enriched in HPP. Anthocyanins, water-soluble flavonoid pigments, participate in plant development and defense and determine fruit sensory and nutritional quality [30]. Among chalcones, 2′,6′-dihydroxy-4,4′-dimethoxychalcone was markedly enriched in HPP, while four other chalcones showed lower accumulation. Chalcones are an important class of polyphenolic compounds belonging to the natural flavonoid and isoflavonoid families, which are widely found in fruits, vegetables, teas, and soy products [31]. In the comparison, flavones were the dominant differential flavonoid subclass, and five of the six anthocyanins accumulated to higher levels in CMP. Similarly to HPP, CMP exhibited strong enrichment of 2′,6′-dihydroxy-4,4′-dimethoxychalcone, whereas several other chalcones preferentially accumulated in YPP. Only a small set of flavonoids differed between CMP and HPP, and most differentially accumulated flavonols were less abundant in CMP, revealing distinct flavonol accumulation patterns between cultivars. For flesh tissues, YPF accumulated higher levels of isoflavones, flavanones, and most chalcones relative to HPF. In CMF, anthocyanins were the main enriched flavonoids compared with YPF, while chalcones, flavanones, and most flavones were more abundant in YPF. When CMF was compared with HPF, anthocyanins and detected chalcones were all depleted in CMF. These tissue-specific metabolic shifts suggest that several metabolites could serve as candidate markers to distinguish these cultivars based on flesh samples.

The distinct flavonoid profiles observed across the three kumquat cultivars may arise from the rewiring of flavonoid biosynthetic flux. Chalcones are open-chain pigmented flavonoids derived from p-coumaroyl-CoA and represent the metabolic entry point into plant flavonoid biosynthesis [32]. One molecule of p-coumaroyl-CoA and three malonyl-CoA molecules are converted to naringenin chalcone (tetrahydroxychalcone, THC) in a reaction catalyzed by chalcone synthase (CHS), the first rate-limiting enzyme of this pathway. THC can be converted to naringenin (flavanone) via chalcone isomerase (CHI); naringenin is further converted to dihydroflavonol by flavanone 3-hydroxylase (F3H). Dihydroflavonol acts as another critical branch point, serving as the shared precursor for flavonols, anthocyanins, and proanthocyanidins [33,34]. Flux distribution from dihydroflavonol toward flavonol or anthocyanin branches is modulated by flavonol synthase (FLS), dihydroflavonol 4-reductase (DFR), and hydroxylase enzymes [35]. Alternatively, chalcone reductase (CHR) competes for CHS reaction intermediates to produce isoliquiritigenin (deoxychalcone), and this diversion of substrate can reduce anthocyanin accumulation, as reported in transgenic petunia overexpressing chalcone reductase 1 (CHR1) [36]. The preferential accumulation of multiple chalcones in YP peel and flesh may indicate that metabolic flux in YP is more inclined toward chalcone retention and flavanone formation. In contrast, HP and CM appear to allocate more metabolic resources toward anthocyanin production in peel tissue.

Through KEGG pathway enrichment analysis, this study systematically explored the differential metabolic regulation between peel and flesh tissues of three kumquat cultivars. In peel tissues, diterpenoid biosynthesis was identified as a major enriched pathway in both the HPP vs. YPP and CMP vs. YPP comparisons. Diterpenoid biosynthesis is closely associated with the accumulation of terpenoid aroma compounds and defense-related secondary metabolites [37,38]. Glycerophospholipid metabolism exhibited the highest enrichment in the CMP vs. HPP pairwise comparison. Indole alkaloid biosynthesis was the primary enriched pathway for the HPF vs. YPF comparison. Histidine metabolism represented the major enriched pathway for the CMF vs. YPF comparison. Histidine is an essential amino acid. Its metabolism primarily occurs through decarboxylation catalyzed by histidine decarboxylase to produce histamine, which is further metabolized into downstream products. Additionally, histidine can undergo transamination to generate glutamate, which participates in energy metabolism, including the tricarboxylic acid (TCA) cycle [39]. Folate biosynthesis was significantly enriched and corresponded to metabolic variation between CMF and HPF. Folate is an essential cofactor in nearly all living organisms, acting as a carrier for one-carbon (C1) units in enzymatic reactions involved in the synthesis of amino acids (methionine, glycine, and serine), purines, thymidylate, pantothenate, and formylmethionyl-transfer RNA [20]. These metabolic features provide a basis for understanding fruit quality differences among the three kumquat cultivars and support the future targeted utilization of kumquat germplasm resources.

This study has several inherent limitations. All fruit samples were harvested from a single orchard at one mature stage; therefore, environmental factors, seasonal variation, and fruit developmental stages may also shape flavonoid profiles and were not evaluated in this work. In addition, metabolite annotation was performed by matching MS/MS fragmentation spectra against public databases, without independent targeted LC-MS validation for most candidate flavonoids. Furthermore, this metabolomic survey only measures metabolite abundance, and cannot clarify the transcriptional regulation underlying the observed metabolic variation. Future work could expand sampling across multiple growing sites and fruit developmental stages to dissect the combined effects of genotype and environment on kumquat flavonoid metabolism. Targeted quantitative analysis of key differentially accumulated flavonoids, together with transcriptome profiling of peel and flesh tissues, will help to uncover the molecular mechanisms governing the tissue- and cultivar-specific flavonoid accumulation. Such multi-omics evidence will assist in screening excellent kumquat germplasm and breeding new cultivars with high nutritional value and tailored flavonoid profiles.

## 5. Conclusions

In this study, UPLC-MS/MS untargeted metabolomics was used to characterize metabolites in the peel and flesh of three Rong’an kumquat cultivars. A total of 5043 metabolites were detected, including 193 annotated flavonoids dominated by flavones and flavonols. Peel tissues possessed more differentially accumulated flavonoid metabolites than flesh tissues. HP- and CM-derived samples (both peel and flesh) exhibited relatively similar flavonoid patterns, while substantial metabolic divergence was observed between the HP–CM and YP samples. The chalcone 2′,6′-dihydroxy-4,4′-dimethoxychalcone was highly accumulated in HP and CM tissues across both peel and flesh, whereas several other chalcone members preferentially accumulated in YP tissues. KEGG enrichment revealed that cultivar variation in peel was associated with diterpenoid and lipid pathways, whereas flesh metabolic divergence was linked to indole alkaloid, histidine, and folate biosynthesis. This study revealed the flavonoid metabolic divergence across different kumquat cultivars and tissues, providing theoretical support for kumquat germplasm screening and quality improvement.

## Data Availability

The original contributions presented in this study are included in the Article/Supplementary Materials. Further inquiries can be directed to the corresponding author.

## Supplementary Materials

The following supporting information can be downloaded at: https://www.mdpi.com/article/10.3390/foods15213934/s1. Table S1: Summary of 5043 LC-MS features detected in three kumquat cultivars; Table S2: Flavonoid metabolites in kumquat; Table S3: Differential flavonoid metabolites from pairwise comparisons of peel and flesh tissues among three kumquat cultivars; Table S4: KEGG annotation of significantly altered metabolites; Figure S1: Quality-control assessment of UPLC-MS/MS data for untargeted metabolomics; Figure S2: Plots of 200-permutation tests for OPLS-DA models.
