# Supplementary material for: Metabolic Characteristics and Flavonoid Differences in Peel and Flesh of Three Kumquat Cultivars Based on Non-Targeted Metabolomics
Source: Foods. 2026 Nov 3;15(21):3934. doi: 10.3390/foods15213934 (PMC13762736; doi:10.3390/foods15213934)
Supplement: Supplementary file 1 [file foods-15-03934-s001.zip › Supplementary figure materials.pdf]

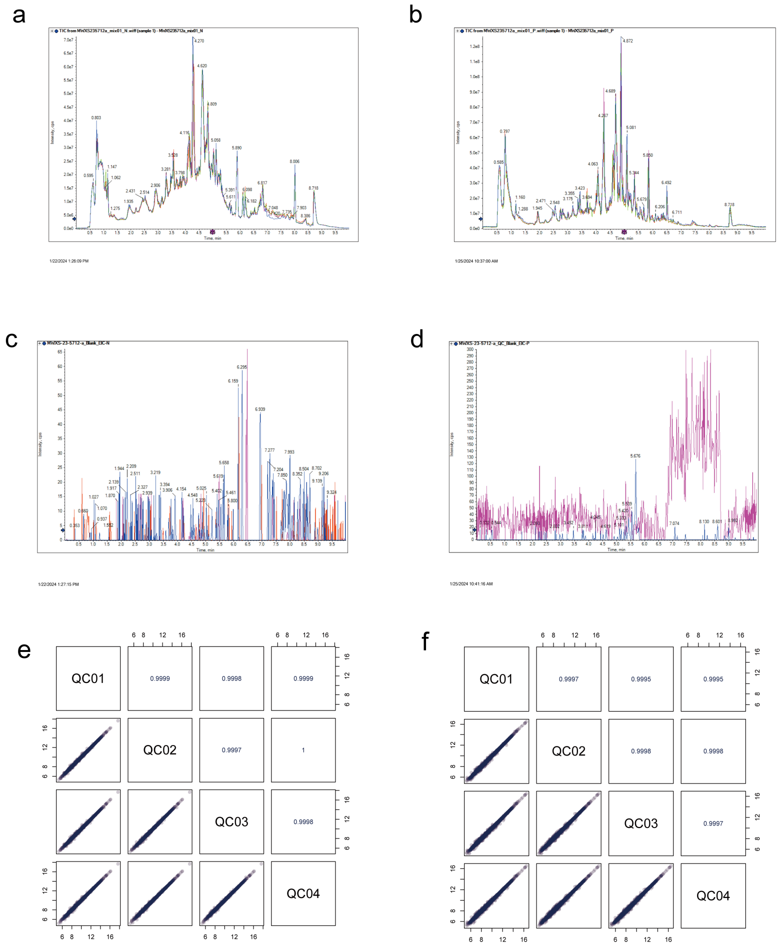

Figure S1. Quality-control assessment of UPLC-MS/MS data for untargeted metabolomics. Overlaid total ion current (TIC) chromatograms (a, b); Extracted ion chromatograms (EICs) of internal standards in blank samples (c, d); Pearson correlation analysis plots of QC samples (e, f). Panels a, c, and e were acquired in positive ionization mode. Panels b, d, and f were acquired in negative ionization mode.

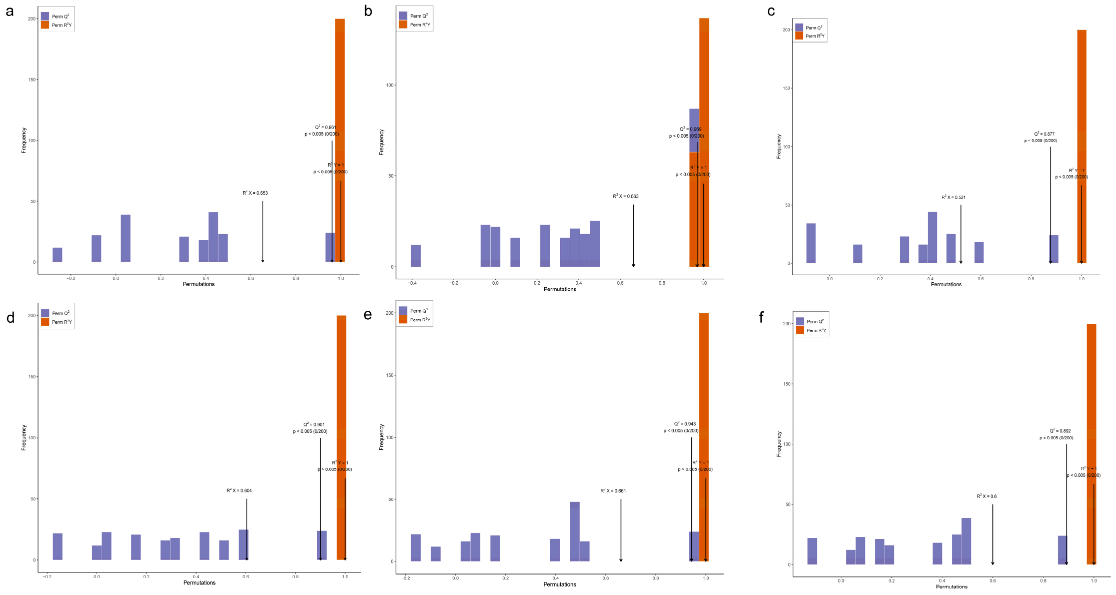

Figure S2. Plots of the 200-permutation tests for the OPLS-DA models. (**a**) HPP vs. YPP; (b) CMP vs. YPP; (**c**) CMP vs. HPP; (**d**) HPF vs. YPF; (**e**) CMF vs. YPF; (**f**) CMF vs. HPF.
